# Steroids, Seizures, and a Surprising Pathogen: A Case of Listeria Meningoencephalitis

**DOI:** 10.7759/cureus.88371

**Published:** 2025-07-20

**Authors:** Shehan M Thangaratnam, May-Lin Wilgus, Grant Turner, Claire E Brown, Kevin Landefeld

**Affiliations:** 1 Department of Internal Medicine, Martin Luther King Community Health, Los Angeles, USA; 2 Department of Medicine, Division of Pulmonary, Critical Care, and Sleep Medicine, University of California Los Angeles, Los Angeles, USA; 3 Department of Medicine, Division of Infectious Diseases, University of California Los Angeles, Los Angeles, USA; 4 Department of Emergency Medicine, University of California Los Angeles, Los Angeles, USA

**Keywords:** asthma, cost of asthma, immunosuppression, inhaler adherence, listeria meningitis, listeria monocytogens, meningitis, poor asthma outcomes, predniso(lo)ne, steroid overuse

## Abstract

Altered mental status is a challenging chief complaint with a wide differential diagnosis. Altered mentation in the setting of new-onset seizures requires consideration of myriad causes, not limited to electrolyte derangements, toxin withdrawals, central nervous system (CNS) infections, hypoxic brain injury, and inflammatory diseases.

We report a 50-year-old man with a history of hypertension and asthma who presented to the emergency department (ED) for new-onset seizures with a prodrome of unusual behavior for several days, and in the ED experienced status epilepticus requiring intubation and deep sedation. He was found to have multiple metabolic derangements, including hypertriglyceridemia, elevated lactate, and diabetic ketoacidosis. Despite correction of initial laboratory derangements, the patient’s mental status did not improve. A lumbar puncture performed on hospital day 3 revealed *Listeria monocytogenes *infection, and the patient subsequently improved slowly with antibiotics.

The culprit microorganism was unexpected. It was later discovered that the patient was taking prednisolone tablets purchased overseas for his asthma because he was unable to afford his asthma maintenance inhalers. This case highlights the challenges in the diagnosis of altered mental status when immune status is unknown, the importance of obtaining collateral history for unprescribed medications, and the dangers of self-medication.

## Introduction

Altered mental status has a wide differential diagnosis, with the unique challenge of necessarily limited history. It requires a thorough physical exam, laboratory testing, and workup with imaging. Analysis of the cerebrospinal fluid (CSF) is often performed if the differential diagnosis includes meningoencephalitis, autoimmune disease, elevated intracranial pressure, or subarachnoid hemorrhage [1].

Bacterial pathogens account for about 10-15% of meningitis and encephalitis cases, with the two most common causes being *Streptococcus pneumoniae* and *Neisseria meningitidis*. *Listeria monocytogenes *(*L. monocytogenes*) usually causes meningoencephalitis rather than strictly meningitis, and accounts for 2-5% of cases in adults. 

*L. monocytogenes* is a gram-positive rod that tolerates a range of temperatures (1-45 °C). It is often introduced from ready-to-eat foods, unpasteurized milk and cheese, and undercooked meats such as chicken, pork, and fish. Its virulence factors include ROS-nullifying listeriolysin O (LLO), anti-phagocytic phospholipases (PLC-A, PLC-B), and pro-motility protein actA [2]. Risk factors for *L. monocytogenes* CNS infection include extremes of age, immunocompromising conditions such as HIV and chronic liver disease, and use of immunosuppressive therapy. It is otherwise rare to find *L. monocytogenes* meningitis in the immunocompetent adult. We aim to highlight the unusual discovery of this unexpected infection and post-hoc immunosuppression status.

## Case presentation

A 50-year-old man with a history of hypertension and asthma presented to the ED with his domestic partner after he was “acting strangely” for five days prior to presentation and had two episodes of “shaking” followed by worsening somnolence. On arrival to the ED, the patient had a witnessed generalized tonic-clonic seizure, prompting midazolam administration and intubation. Vital signs were significant for tachycardia to 156 bpm, and hypertension to 183/98 mmHg. Physical exam demonstrated a sedated, obese male with thinning hair and a protuberant abdomen. No nuchal rigidity was noted. The patient’s labs (Table 1) were significant for leukocytosis, hyponatremia, hypertriglyceridemia, with elevated glucose, ketones, and low bicarbonate, pseudohyponatremia as evidence of diabetic ketoacidosis. He was started on an insulin drip, bicarbonate drip, empiric antibiotics (vancomycin and piperacillin/tazobactam), and midazolam for sedation with improvement in initial laboratory derangements. On hospital day 2, he became febrile to 40 °C and required increasing doses of midazolam for ongoing seizures.

**Table 1:** Serum values on admission WBC = white blood cell count; Na = sodium; Cr = creatinine; CO₂ = carbon dioxide; TG = triglycerides; Glc = glucose

Parameters	Patient Values on Admission	Reference Range
WBC	12.0 x 103/ μL	4.1–9.9 x 103/ μL
Na	125 mmol/L	135–146 mmol/L
Cr	2.30 mg/dL	0.6–1.3 mg/dL
CO_2_	9 mmol/L	20–30 mmol/L
Lipase	101 U/L	13–69 U/L
TG	1570 mg/dL	<150 mg/dL
Glc	330 mg/dL	65–99 mg/dL
Ketone	Positive	-

Neurology was consulted, who advised further workup with a brain MRI that showed no acute abnormality. Nephrology was consulted, and the patient was started on intermittent hemodialysis for metabolic acidosis. Despite aggressive medical management. On hospital day 3, the patient had worsening circulatory shock requiring norepinephrine for hypotension, and a lumbar puncture was performed to evaluate for meningitis (Table 2), which prompted Infectious Diseases consultation. The CSF culture remained negative for 48 hours; however, the meningoencephalitis PCR panel detected* L. monocytogenes* - the same day the CSF culture returned positive with corroborating results. Antibiotics were adjusted to ampicillin and gentamicin, with guidance from the Infectious Disease service, with a gradual improvement in seizure activity. He began to intermittently follow commands, and on hospital day 6, corresponding to day 3 of appropriate antibiotic coverage, the patient was successfully extubated to high-flow nasal cannula. He ultimately completed a three-week course of ampicillin.

**Table 2:** Results of CSF analysis and related microbiology culture data CSF = cerebrospinal fluid; TNC = total nucleated count

CSF	Value	Reference Range
Glucose	96 mg/dL	50 - 80 mg/dL
Protein	327 mg/dL	18 - 60 mg/dL
Cell count/differential	207 TNC/μL	-
Culture	Positive for *L. monocytogenes *	-
Meningoencephalitis panel	Positive for *L. monocytogenes *	-

Initial history obtained from the patient’s partner was limited; however, collateral information later obtained from the patient’s parents revealed that he had been purchasing prednisolone 20 mg from overseas. He was reported to take 1-3 tablets (20 to 60 mg) daily for the past two years for his asthma at the advice of a friend who was not a medical provider. In review of outpatient clinic visits over the preceding three years, the patient reported often being unable to afford the cost of asthma maintenance medication (fluticasone/salmeterol). Oral steroids were not obtained from his primary care physician but were purchased online from a source in India.

## Discussion

Bacterial meningitis is a well-discussed, yet infrequent illness with a high morbidity, with an incidence of 0.9 per 100,000 individuals and a mortality rate of up to 50% [3]. The common presenting symptoms of fever, headache, neck stiffness, and altered mental status typically prompt CSF analysis and culture, the gold standard for diagnosis, along with multiplex PCR, which may be especially valuable if the patient has been treated with antibiotics prior to undergoing lumbar puncture. Our patient lacked the classic constellation of symptoms and did not have signs of neck stiffness on exam. However, only 40-50% of patients present with the full constellation of symptoms, and classic physical exam findings of Kernig and Brudzinski signs have reported sensitivity of only 5-30% [4]. Community-acquired bacterial meningitis presents with seizures in 17% of patients, with seizure-positive cases of bacterial meningitis at a much higher likelihood of death [5]. When bacterial meningitis is suspected, IV antibiotics and steroids should be administered without delay. Empiric antibiotic treatment typically includes vancomycin and a third-generation cephalosporin, with the addition of ampicillin in patients above 50 years of age, under a year of age, or immunosuppressed (this patient’s immune suppression was not initially known). Early dexamethasone administration within the first four days of treatment has been found to reduce adverse outcomes and mortality in adult patients with bacterial meningitis [6]; however, less data is available for patients who were not treated for meningitis within the first day of hospitalization. Furthermore, it has been commonly advised to stop steroid therapy for confirmed *L. monocytogenes* meningitis [4]. However, a 2023 French cohort study demonstrated less favorable outcomes, including increased mortality, in Listeria meningitis patients who did not receive standard dexamethasone [7].

Patients with *L. monocytogenes* can have a low-grade fever and subtle personality changes in the early stages of infection; however, once fulminant *L. monocytogenes* is indistinguishable from other causes of bacterial meningitis [8]. This case is a classic example of *L. monocytogenes* meningitis in an unexpected host, who was found post-hoc to be immunocompromised due to non-prescribed medication. The patient’s obese body habitus, diabetes mellitus, hypertriglyceridemia, and hypertension are explained by his occult prednisolone use.

While this patient had neurological recovery and was discharged on room air to a rehabilitation center, he had a prolonged hospital course including intermittent hemodialysis, permanent kidney dysfunction with a new baseline of Cr 3.5 mg/dL, and repeat hospitalizations for fluctuating encephalopathy/syncope with intermittent short-term memory loss. He was counseled to refrain from taking medications not prescribed by his medical providers.

Undertreatment of asthma and its costs

Individuals taking oral steroids daily or even intermittently for the treatment of asthma are at risk for dose-dependent adverse effects, including infection, diabetes, and osteoporosis [9]. Inhaled corticosteroid (ICS) therapy has become increasingly central to the management of asthma at all stages, especially as it shows greater efficacy than β2-agonists for adults and children, and spares exposure to oral steroids [10]. However, oral steroid self-medication and its inappropriate use do occur in people with obstructive airway diseases, though numerical data are elusive [11]. In this case, an underinsured patient encountered high costs for asthma inhaler treatment and resorted to prednisolone obtained overseas as an alternative, unaware of the risks of chronic systemic steroid use. He was met with a very rare infection as an adverse effect, with far more costly ramifications.

Asthma is a common disease of adulthood that affects approximately 5.4% of the global population, and uniquely, it has a higher proportion of medication nonadherence due to medication costs than other chronic diseases [12]. The estimated cost of asthma in the US is estimated to be as high as $80 billion annually for “treated asthma” [13], and this number can balloon to $300 billion annually owing to increased healthcare visits, hospital admissions, and lost productivity [14]. Asthma management encompasses appropriate assessment of a patient’s symptom burden, medication efficacy, and long-term adherence [15]. Medication adherence in asthma is reported as low as 30%, and medication cost is a frequently identified barrier [16, 17]. In a large cross-sectional study of over 26,000 asthmatics, 16% reported cost-related medication non-adherence [18], affecting both uninsured and “underinsured” individuals with large out-of-pocket costs. 

## Conclusions

We present a case of *L. monocytogenes* meningoencephalitis in a patient who was found to be immunocompromised due to the use of oral prednisolone obtained on the internet, as he was unable to afford asthma controller inhaler therapy. Physicians should continue to maintain a high degree of suspicion for meningitis and evaluate promptly and consider broad antimicrobial coverage with CNS penetration in ill-appearing patients with seizures and encephalopathy. Physicians should also be aware of the availability of unregulated overseas pharmaceuticals that may tempt patients who face high prescription drug costs in the United States.
